# WNK1 and p38-MAPK distribution in ionocytes and accessory cells of euryhaline teleost fish implies ionoregulatory function

**DOI:** 10.1242/bio.024232

**Published:** 2017-05-18

**Authors:** W. S. Marshall, R. R. F. Cozzi, M. Spieker

**Affiliations:** Biology Department, St. Francis Xavier University, 2320 Notre Dame Avenue, Antigonish, Nova Scotia B2G 2W5, Canada

**Keywords:** WNK1, p38MAPK, NKCC, CFTR, Chloride cell, Accessory cell, *Fundulus heteroclitus*, Teleost fish, Freshwater, Seawater, Hypersaline, Immunocytochemistry, Forskolin

## Abstract

Ionocytes of euryhaline teleost fish secrete NaCl, under regulation by serine and threonine kinases, including with-no-lysine kinase (WNK1) and p38 mitogen-activated protein kinase (MAPK). Mummichogs (*Fundulus heteroclitus* L.) were acclimated to freshwater (FW), full strength seawater (SW) and hypersaline conditions (2SW). Immunocytochemistry of ionocytes in opercular epithelia of fish acclimated to SW and 2SW revealed that WNK1-anti-pT58 phosphoantibody localized strongly to accessory cells and was present in the cytosol of ionocytes, close to cystic fibrosis transmembrane conductance regulator (CFTR) in the apical membrane and the sodium potassium 2 chloride cotransporter (NKCC) in the basolateral membrane. In FW acclimated fish, WNK1 localized to a sub-apical zone, did not colocalize with apical membrane-located sodium chloride cotransporter (NCC), and typically was present in one cell of paired ionocytes and in some single ionocytes. Forskolin treatment (10 μM, 30 min) increased WNK1 immunofluorescence in SW ionocytes only, while hypertonicity had little effect, compared to controls. Anti-p38-MAPK antibody localized to the cytosolic compartment. The distribution of WNK1 and p38MAPK is consistent with a proximal position in regulatory cascades, rather than directly affecting transporters. The strong staining of accessory cells by WNK1 phosphoantibody infers an osmoregulatory function for WNK.

## Abstract (precis)

**Summary:** Fish opercular epithelium ionocytes and accessory cells have WNK family kinases that may regulate paracellular and transcellular ion transport.

## INTRODUCTION

The ionocytes (formerly known as mitochondrion-rich cells and chloride cells) of teleost fish are a focus of research into osmoregulation and ion balance (Edwards and Marshall, 2013; Evans et al., 2005). In marine teleost fish and euryhaline teleost fish acclimated to full strength seawater (SW), ion secretion is accomplished by ionocytes via a basolateral-located sodium potassium 2 chloride cotransporter, NKCC1, which drives Cl^−^ into the cell, in series with the anion channel cystic fibrosis transmembrane conductance regulator (CFTR) in the apical membrane, by which Cl^−^ leaves down its electrochemical gradient. In parallel, Na^+^ is secreted by way of a paracellular pathway that is cation selective and exists in special tight junctions between ionocytes and smaller mitochondrion-poor accessory cells (Sardet et al., 1979). The opercular epithelium is the most practical tissue for examining the fine structure of ionocytes and accessory cells, which are functionally and morphologically similar to the ionocytes of the gill epithelium.

Regulation of the transcellular ion transport pathway involves hormone and neurotransmitter regulation, principally via Ca^2+^-mediated inhibition of Cl^−^ secretion and cyclic adenosine monophosphate (cAMP)-mediated stimulation of Cl^−^ secretion via activation of NKCC and CFTR. In addition, ionocytes are osmosensitive, and transcellular Cl^−^ secretion is inhibited by hyposmotic swelling and stimulated by hyperosmotic cell shrinkage. We know that volume sensitivity involves integrin α/β stretch receptors with downstream events involving focal adhesion kinase (FAK), stress-activated protein kinase (JNK1), oxidative stress response kinase (OSR1), p38 mitogen-activated protein kinase (p38MAPK) and proline-alanine-rich ste20-related serine/threonine protein kinase (SPAK) regulation of basolateral NKCC (Flemmer et al., 2010; Hoffmann et al., 2007; Marshall et al., 2005). Furthermore, FAK phosphorylates during hypertonic shock and dephosphorylates during hypotonic shock in seawater ionocytes, and is colocalized with NKCC and CFTR (Marshall et al., 2009). FAK can apparently also regulate CFTR at the apical membrane (Marshall et al., 2009), and in mammalian airway epithelial cells (Calu-3) the related protein tyrosine kinase-2, PYK2, activates CFTR (Billet et al., 2015). In mammalian systems, with-no-lysine kinase (WNK1) is also involved in ion transport regulation (see below), and the present research attempts to connect WNK1 to transport regulation in teleost ionocytes by localizing this regulatory enzyme to the ion transporting cells.

WNK forms a family (WNK1-4) of serine/threonine kinases known to upregulate and activate cation-chloride transporters, such as NKCC1, but indirectly by activation of more distal kinases (e.g. SPAK, and OSR1) in regulatory cascades (Delpire and Gagnon, 2006, 2008, Sengupta et al. 2012). In mammalian cells, WNK4 is commonly associated with OSR1 and SPAK, and chimeras across animal phyla in frog oocytes also phosphorylate NKCC, suggesting broad evolutionary conservation of this signaling pathway (Gagnon et al., 2011). The pathway is associated with cell volume regulation, specifically regulatory volume increase via NKCC activation (Delpire and Gagnon, 2008). WNK1 is inhibited by high chloride concentration (Piala et al., 2014), allowing the hypothesis that the transcellular chloride transport that occurs in numerous salt secreting epithelia across vertebrates (e.g. in shark rectal glands, avian salt secretion, reptilian salt secretory glands and marine teleost fish gills), may potentially share a chloride-sensing regulatory pathway to autoregulate NaCl secretion rate. Regulation of the sodium chloride cotransporter (NCC) has recently been shown to include WNK and SPAK (Pacheco-Alvarez et al., 2012), suggesting that epithelia relying on NCC for NaCl uptake, such as in freshwater (FW) teleost fish, may also use this pathway.

The role of accessory cells is poorly understood. Accessory cells in SW teleost fish have few mitochondria (Hootman and Philpott, 1980; Laurent et al., 2006) and fail to stain strongly for Na^+^/K^+^-ATPase, NKCC or CFTR, so it is unlikely that they are important in active Cl^−^ secretion, compared to the larger, mitochondrion-rich ionocytes that are strongly positive for all three of the previously mentioned proteins. Initially accessory cells were thought to be degenerate forms of chloride cells (Hootman and Philpott, 1980) and it has been suggested that, in FW acclimated Mozambique tilapia (*Oreochromis mossambicus*, Peters, 1852), similarly positioned accessory cells were immature mitochondria-rich ionocytes (Bonga et al., 1990). Early and recent observations in seawater acclimated fish noted the close association of accessory cells with the cation-permeable junctions at this location (Laurent et al., 2006; Sardet et al., 1979) and implied the involvement of accessory cells with tight junctions. It was further suggested that accessory cells may send processes to interact with the ionocytes (Laurent et al., 2006). Additionally, more elaborate interactions between accessory cells and ionocytes develop when Nile tilapia (*Oreochromis niloticus* L.) are fed high salt diets (Fontainhas-Fernandes et al., 2000) and in mummichogs acclimated to hypersaline conditions (Cozzi et al., 2015), suggesting an active role of accessory cells in regulation of the paracellular pathway in seawater and the elaboration of those junctions in hypersaline conditions. The relationship appears to be limited to seawater and hypersaline conditions (2SW), as FW acclimated Nile tilapia have similarly positioned cells adjacent to ionocytes that were termed ‘support cells’, rather than accessory cells, as the intercalation/interdigitation structures were absent (Monteiro et al., 2010). In FW brown trout (*Salmo trutta* L.) larvae and juveniles, the appearance of alpha and beta mitochondrion-rich ionocytes are also paired with ‘accessory cells’ (Pisam et al., 2000), but these cell types may represent functional pairs of Na^+^ and Cl^−^ uptake ionocytes specialized for ion uptake from FW (Dymowska et al., 2012; Kumai and Perry, 2012; Tresguerres et al., 2006), or ionocytes specialized for calcium uptake from FW (Hsu et al., 2014). So the function of seawater accessory cells and their regulatory relationship with seawater ionocytes remains enigmatic.

The present work demonstrates that WNK1 is present in ion transporting cells in fish acclimated to FW, SW and 2SW; is distributed in cytoplasmic zones that do not exactly colocalize with transporters; is activated by forskolin; and, interestingly, appears very prominently in seawater accessory cells, so much so that this indicator could be used to specifically stain accessory cells.

## RESULTS

### Immunocytochemistry

#### SW and 2SW acclimated mummichogs

##### WNK1 and NKCC

**Fig. 1.**
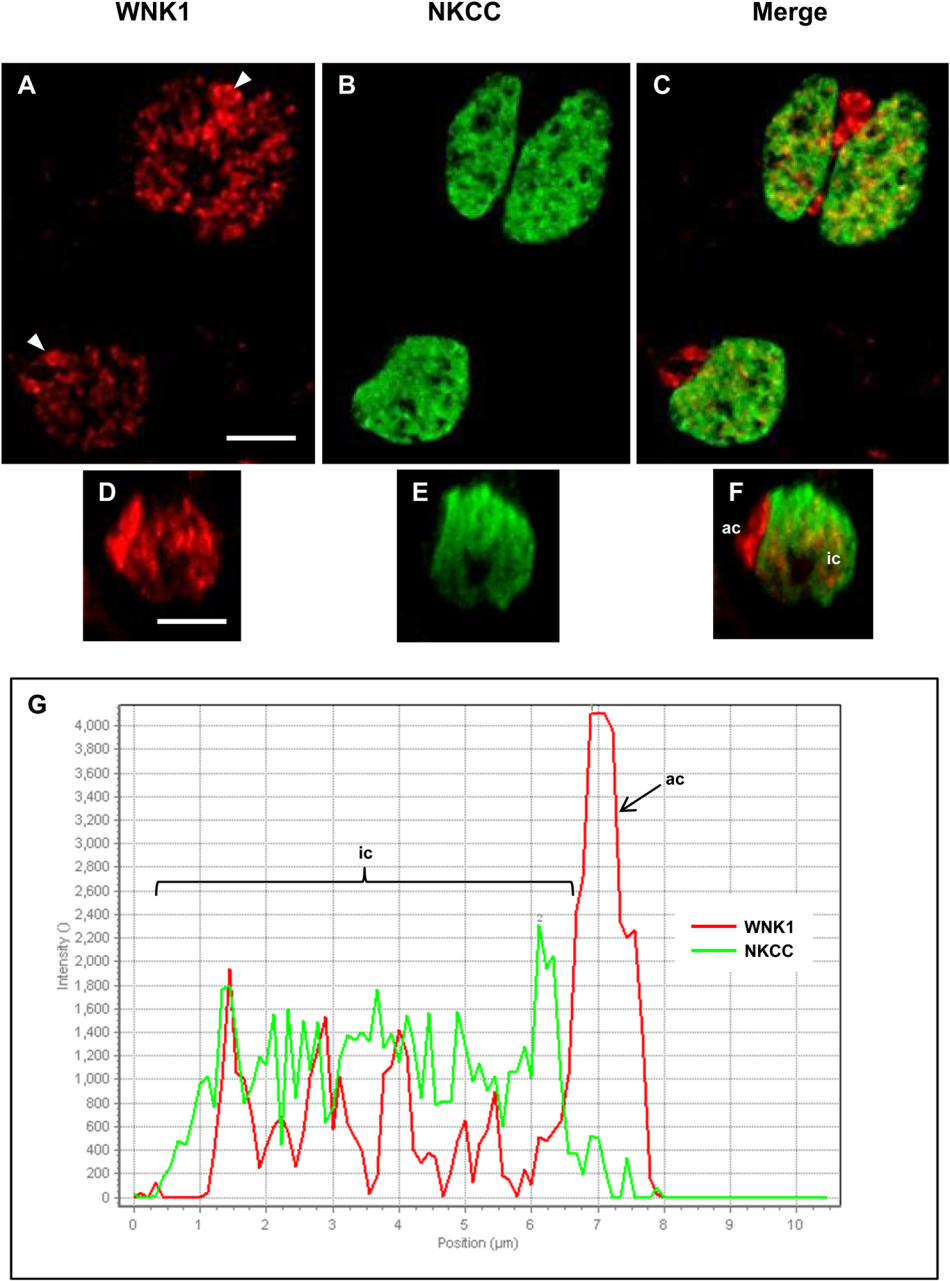
**WNK1 and NKCC immunofluorescence in SW mummichog OE.** (A) WNK1 (red) was distributed in ionocytes and had a stronger immunofluorescence in the accessory cells (arrowheads). (B) NKCC (green) was present in ionocytes, but absent from accessory cells. (C) Merged image of frames A and B showing that WNK1 and NKCC are located within 1 μm in ionocytes, but not co-localized. (D) An *xz*-scan, showing WNK1 immunofluorescence in an ionocyte and accessory cell (side view). (E) Same frame as D, but NKCC immunofluorescence. (F) Merged image of D and E. (G) Line scan across an ionocyte showing WNK1 and NKCC immunofluorescence intensity (arbitrary units) in the ionocyte (ic) and accessory cell (ac), with ∼two-fold higher intensity WNK1 immunofluorescence in the accessory cell than in the ionocyte. *n*=10. Scale bars: 5 µm.

**Fig. 2.**
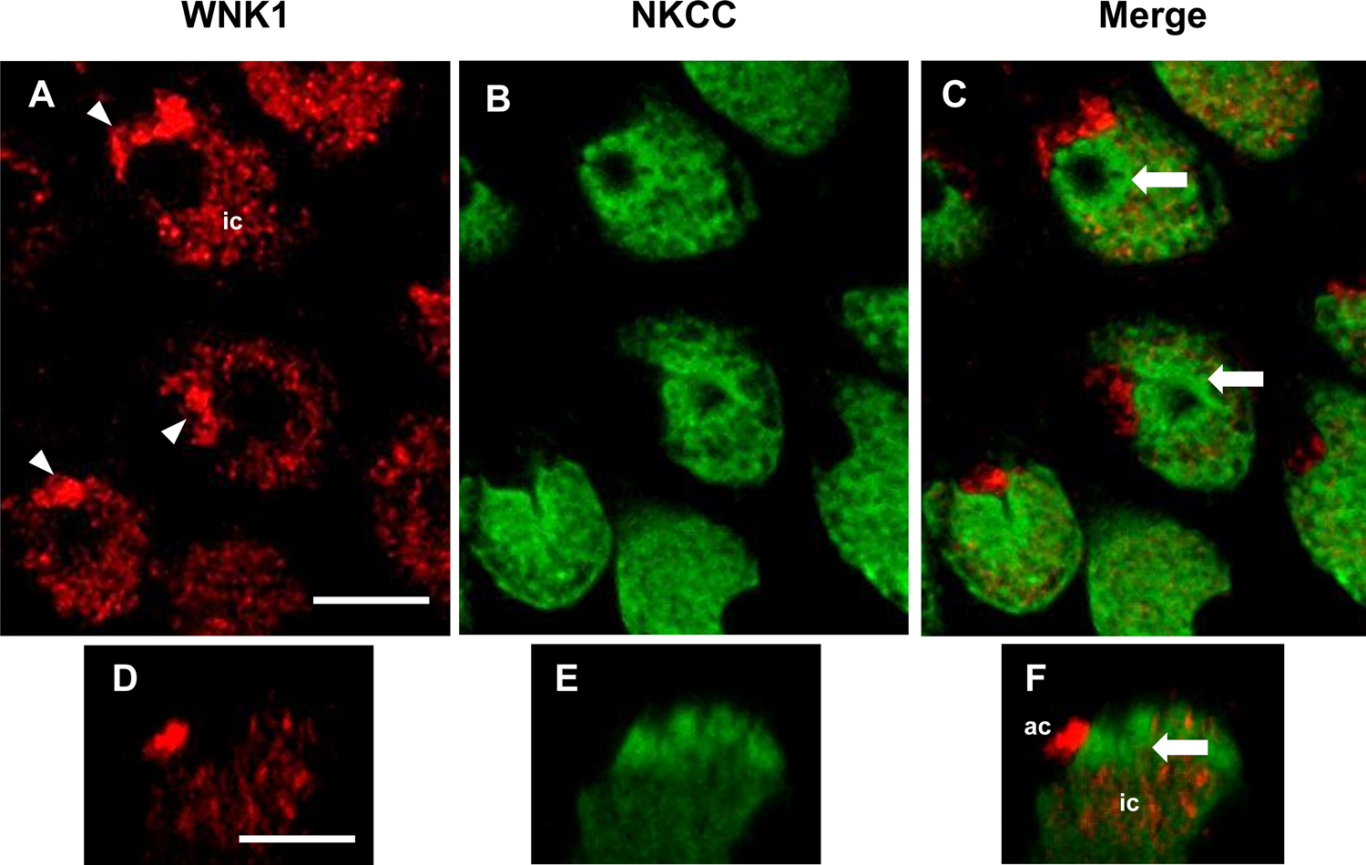
**WNK1 and NKCC immunofluorescence in hypersaline-exposed 2SW mummichog OE.** (A) WNK1 (red) was distributed in ionocytes (ic) and had stronger immunofluorescence in the accessory cells (ac, arrowheads). (B) NKCC (green) was present throughout the ionocytes, but was absent from accessory cells. (C) Merged image of frames A and B showing the absence of WNK1 and NKCC colocalization in ionocytes. NKCC was present in the space surrounding the apical crypt area, whereas WNK1 was absent (arrows). (D) An *xz*-scan, showing WNK1 immunofluorescence in an ionocyte and accessory cell (side view). (E) Same frame as D, but NKCC immunofluorescence. (F) Merged image of D and E. *n*=6. Scale bars: 5 µm.

WNK1 was detected in SW and 2SW mummichog opercular epithelia (OE); WNK1-pT58 immunofluorescence was observed in ionocytes, but was more prevalent in accessory cells, appearing brighter (Fig. 1A,C) and of a two- to five-fold higher immunofluorescence intensity (Fig. 1G), than in the ionocytes. All SW (Fig. 1) and 2SW (Fig. 2) ionocytes possessed WNK1 and NKCC. NKCC immunofluorescence was evenly distributed in the ionocytes, consistent with localization in the tubular system, whereas WNK1 immunofluorescence was observed in clusters throughout the accessory cells. However, there was no colocalization between the two; NKCC was also never present in the accessory cells (Fig. 1C,F,G and Fig. 2C,F).

##### WNK1 and CFTR

**Fig. 3.**
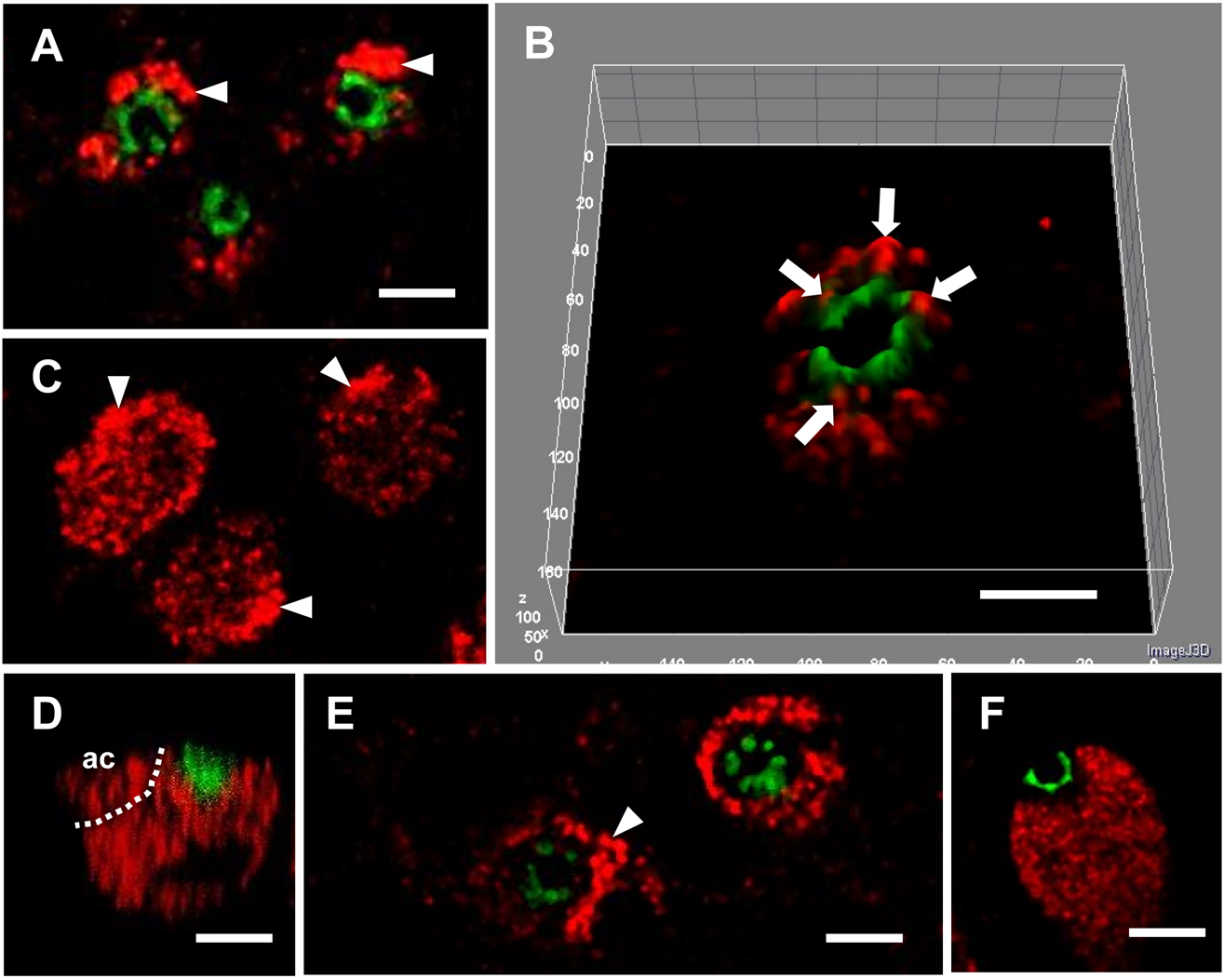
**WNK1 and CFTR immunofluorescence in SW and 2SW mummichog OE.** (A-D) SW, (E,F) 2SW. (A) In SW OE, 2 µm below the surface, WNK1 (red) was present in ionocytes and had stronger immunofluorescence in the accessory cells (arrowheads). CFTR (green) was only present in the apical crypt. (B) A 3D surface plot showing the close proximity of WNK1 and CFTR in the apical crypt (arrows), and the absence of colocalization; CFTR distribution is ring-like with one gap on the left side (where an accessory cell is present). (C) Same image as A, but 6 µm below the surface, confirming WNK1 immunofluorescence in deeper parts of the ionocytes and the accessory cells. (D) An *xz*-scan, showing a SW ionocyte and accessory cell (ac) stained strongly for WNK1. (E) WNK1 immunofluorescence in OE from fish acclimated to 2SW was the same as in fish acclimated to SW. CFTR distribution was punctate (concentrated into dots). An apparent exclusion zone was visible between the CFTR-positive apical crypt and areas of WNK1 immunofluorescence. (F) An *xz*-scan showing the side view of a 2SW ionocyte, where WNK1 was present deeper in the ionocyte below an apparent exclusion zone. *n*=8. Scale bars: 5 µm.

In SW acclimated fish, CFTR was evenly distributed in the apical crypt of ionocytes (Fig. 3A,B) and WNK1 was found in close proximity to the apical crypts, but did not colocalize with CFTR (Fig. 3A,B,D). Accessory cells stained brightly for WNK1 (Fig. 3A,C,D). Acclimation to 2SW changed CFTR distribution such that CFTR immunofluorescence formed a beaded appearance (Fig. 3E,F) and there was an apparent withdrawal of WNK1 immunofluorescence surrounding the apical region, thus creating a gap between the apical crypt CFTR and WNK1 immunofluorescence in the ionocytes (Fig. 3E,F). NKCC was present in the space between the apical crypt and WNK1 [compare Fig. 2A,C (arrows) with Fig. 3E,F]. Analysis of the proximity of WNK1 to CFTR revealed a significant difference in the distance between the apical crypt and WNK1 in SW and 2SW ionocytes. WNK1 was detected significantly deeper in 2SW ionocytes (0.75±0.03 µm) compared to SW ionocytes (0.24±0.02 µm) (***P<0.001 by unpaired *t*-test, values are mean±s.e.m., *n*=8 epithelia).

#### FW acclimated mummichogs

##### WNK1 and NCC

**Fig. 4.**
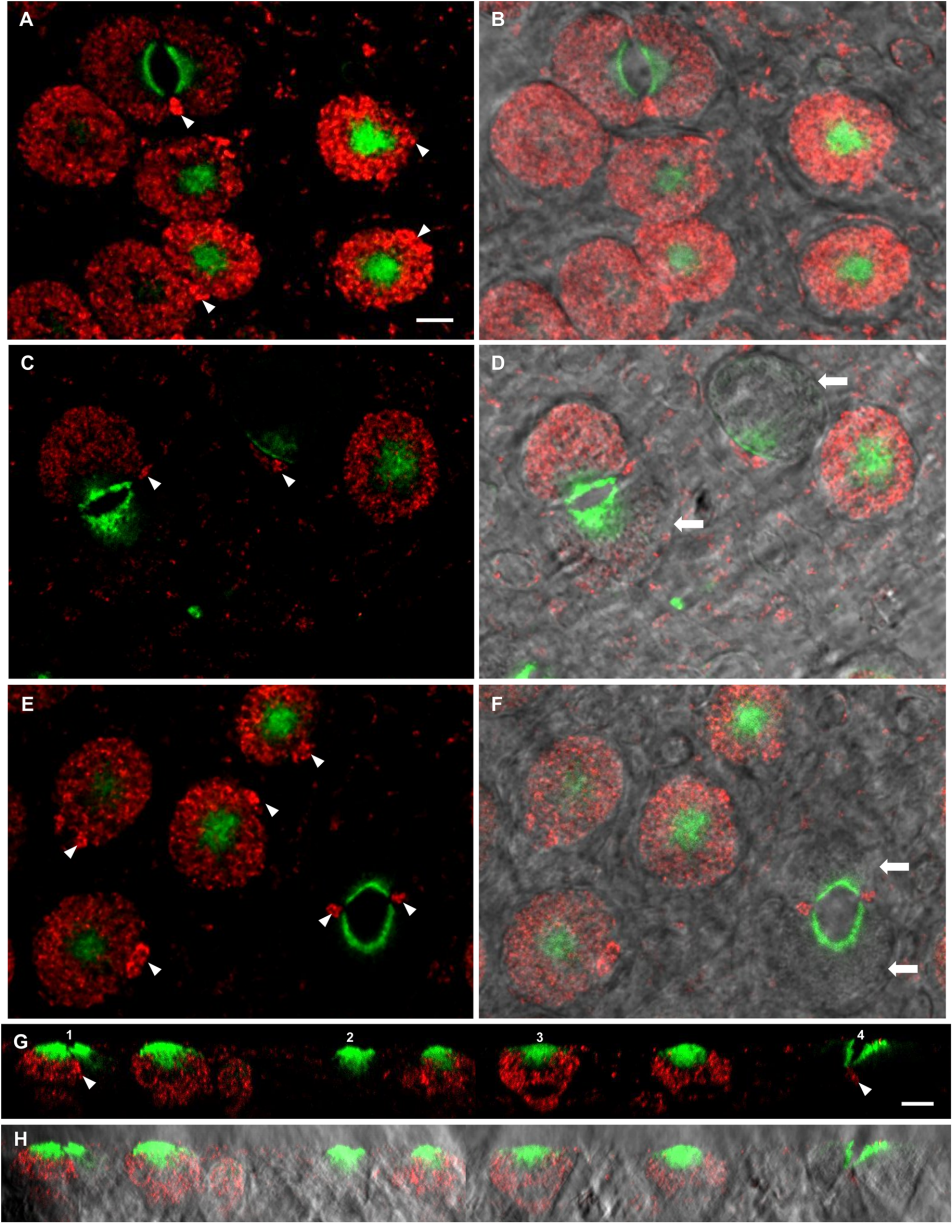
**WNK1 and NKCC immunofluorescence in FW mummichog OE.** (A) WNK1 (red) was mostly distributed in single and paired ionocytes. Immunofluorescence was stronger in the accessory cells (arrowheads). Immunofluorescence, likely NCC rather than NKCC (green), was localized in the apical area of the ionocytes and was absent from the accessory cells. In paired cells, immunofluorescence was located in the cytosol near the shared surface of the two ionocytes. Accessory cells were also present between paired ionocytes. (B) Same frame as A, with bright field. (C) Although WNK1 immunofluorescence was present in all accessory cells (arrowheads), it was sometimes absent from single ionocytes and only present in one of the two cells of paired ionocytes. (D) Same frame as C, with bright field, showing WNK1-negative ionocytes (arrows). (E) Single and paired ionocytes with WNK1-positive accessory cells. In some paired cells, WNK1 was absent from both ionocytes in the pair. (F) Same frame as E, with bright field, showing a WNK1-negative ionocyte pair (arrows). (G) Merged image of WNK1 and NCC immunofluorescence (*xz*-scan), showing distinct distribution and the absence of colocalization in ionocytes. NCC immunofluorescence was located only in the apical area of ionocytes; WNK1 immunofluorescence was present in all accessory cells (arrowheads), but not in all ionocytes. G1, ionocyte pair with one WNK1-positive and one WNK1-negative ionocyte; G2, single WNK1-negative ionocyte; G3, single WNK1-positive ionocyte; G4, ionocyte pair with both WNK1-negative cells. (H) As G, with bright field. *n*=8. Scale bars: 5 µm.

**Fig. 5.**
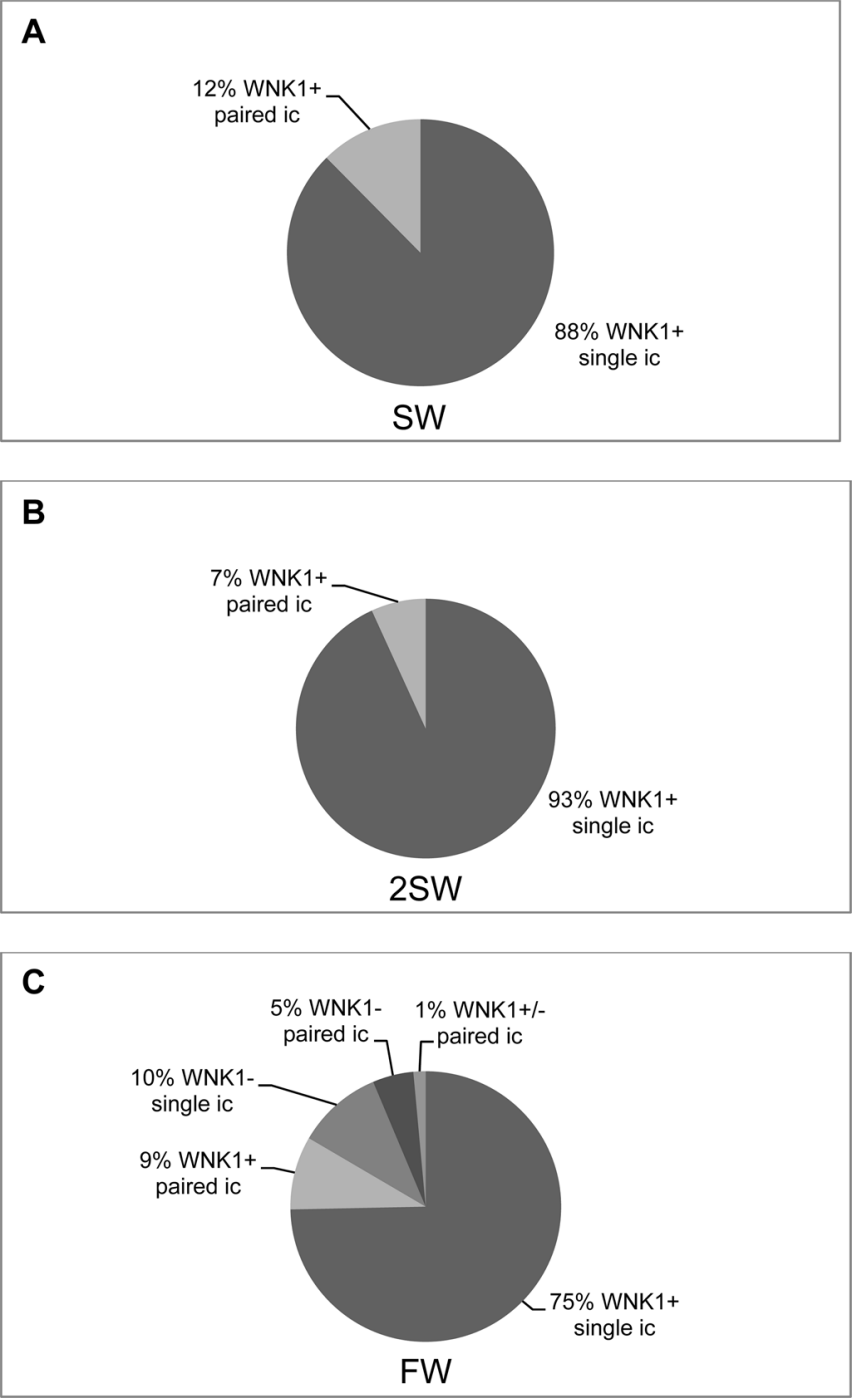
**Analysis of WNK1 distribution in SW, 2SW and FW ionocytes.** (A) WNK1 was present in all SW ionocytes, single (88%) and paired (12%). (B) WNK1 was also present in all 2SW ionocytes (single 93%, paired 7%). (C) In FW, WNK1 distribution varied. The majority of the single ionocytes were WNK1 positive (75%). However, 10% of the single ionocytes identified by NKCC staining were WNK1 negative. In the paired ionocytes (15% of the total), 5% were WNK1 negative in both cells, 9% were WNK1 positive in both cells, and 1% had WNK1 in only one of the two ionocytes. *n*=6.

WNK1 distribution varied in ionocytes of FW acclimated fish (Fig. 4). Most ionocytes were WNK1 positive (Fig. 4A-F), but some ionocytes that were positive for the T4 antibody (which presumably represents NCC protein rather than NKCC), were not stained for WNK1 (arrows, Fig. 4D,F,G1,G2). WNK1 was present in small accessory cells (arrowheads, Fig. 4A,C,E,G1). In FW, ionocytes were also noticeably larger (compare those in Fig. 4A,B to SW ionocytes in Fig. 1) and NCC immunofluorescence was re-localized to the apical membrane in the ionocytes (Fig. 4G,H). The ionocytes often formed pairs in which NCC was localized to crescent-shaped apical areas in which the two ionocytes were connected and shared an accessory cell (Fig. 4E,G1,G4). There was no colocalization of WNK1 with NCC (Fig. 4G3). CFTR immunofluorescence was completely absent from FW ionocytes (data not shown). An analysis of WNK1 distribution in FW ionocytes revealed that WNK1 distribution varied in both types of cell arrangement (Fig. 5). Although 84% of the ionocytes (75% single cells and 9% paired cells) possessed WNK1, 15% of ionocytes (10% single cells and 5% paired cells) were WNK1 negative. Furthermore, in some cases (1%), paired ionocytes possessed WNK1 in only one of the two cells (Fig. 4D,G1). The FW distribution suggests multiple cell types, some WNK positive and others WNK negative, whereas a similar analysis of SW and 2SW cells (Fig. 5) revealed that in both types of cell arrangement, single cells (SW 88% and 2SW 93%) and paired cells (12% and 7%, respectively) were positive for WNK1.

#### Forskolin and hypertonicity

**Fig. 6.**
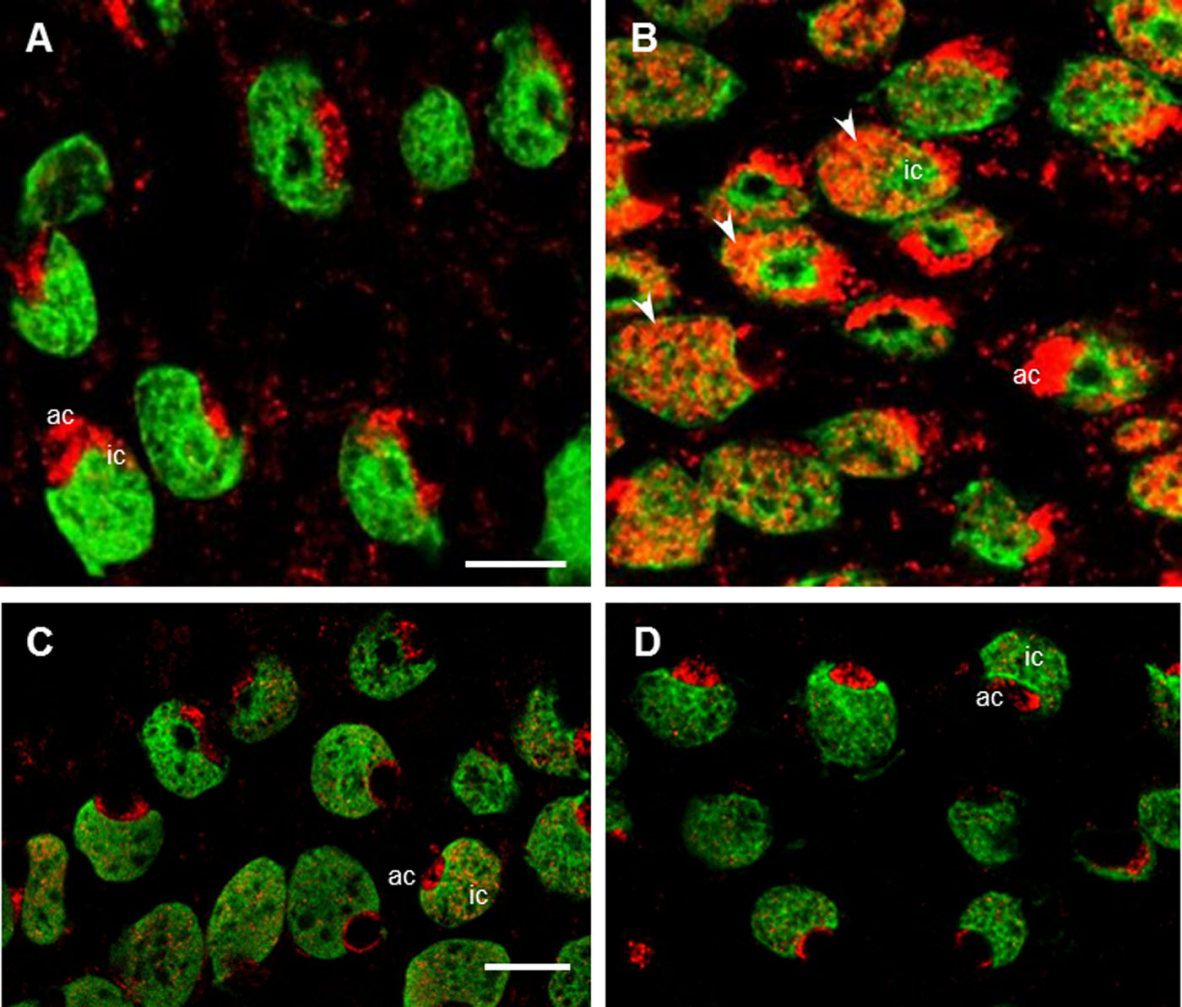
**Effects of forskolin and hypertonicity on WNK1 phosphorylation in ionocytes and accessory cells.** WNK1 (red) and NKCC (green) immunofluorescence in (A) control SW OE and (B) forskolin-treated SW OE (10 μM 30 min). Note the higher WNK1 signal in most ionocytes (arrowheads). *n*=2. Scale bar: 10 μm. WNK1 (red) and NKCC (green) immunofluorescence in (C) control SW OE and (D) SW OE after hypertonic treatment (30 min, Cortland's saline ^+^42 mM NaCl). Hypertonicity had little effect on WNK1 immunofluorescence. *n*=4. Scale bars: 10 μm.

To test for activation of WNK1, OE from SW acclimated fish were incubated in aerated Cortland's saline with 10 μM forskolin and compared to control tissues without the drug. Immunocytochemistry for WNK1-pT58 revealed increased fluorescence in ionocytes relative to controls, and compared to NKCC fluorescence, which was constant (Fig. 6A,B). Accessory cells also appeared to have an increased WNK1 signal, but this was less obvious, as the accessory cells of controls were already brightly fluorescent. Hypertonicity with mannitol and NaCl addition increases NaCl secretion rate (Marshall et al., 2005), and hypertonicity by adding NaCl (^+^42 mM final concentration) to Cortland's saline increased WNK1 fluorescence noticeably, compared to controls without added NaCl (Fig. 6C,D).

#### Actin and WNK1

**Fig. 7.**
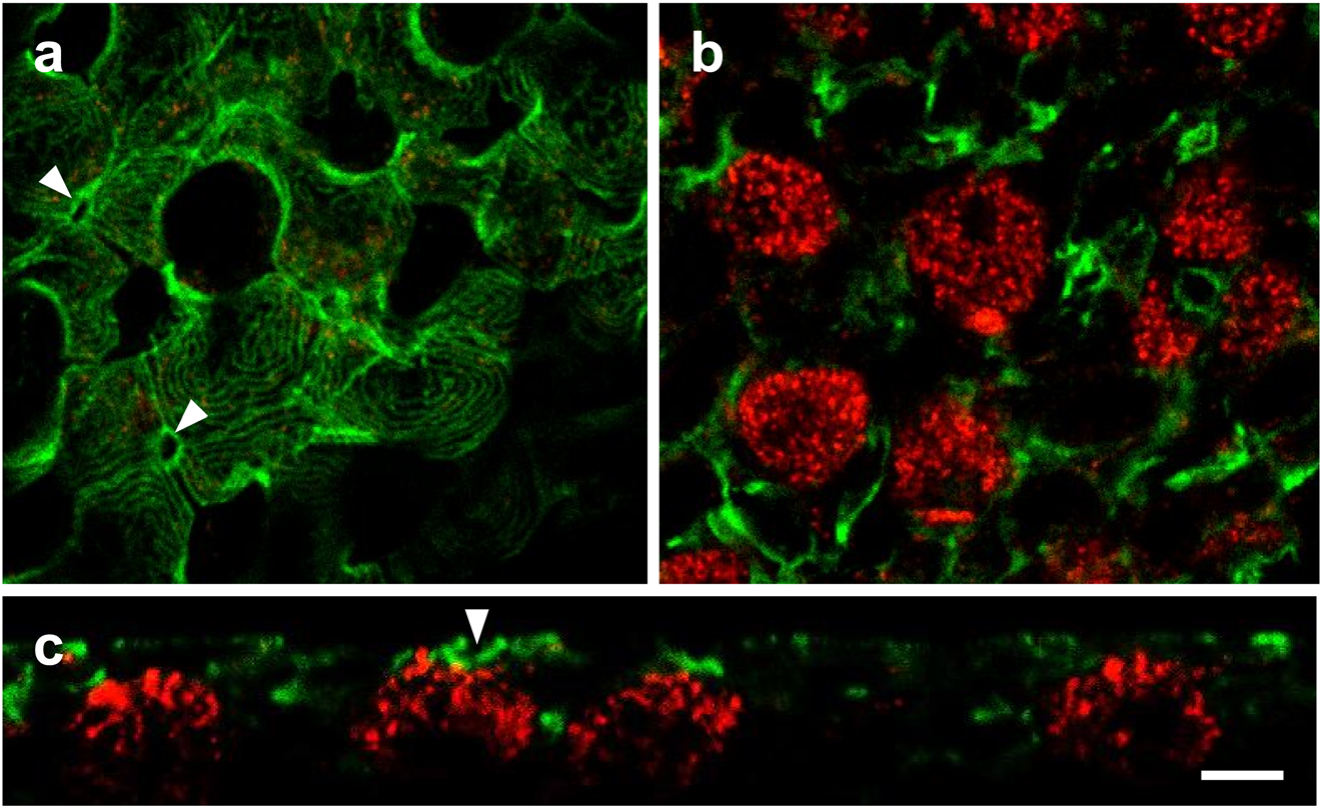
**WNK1 and actin distribution in SW OE.** (A) WNK1 (red) and actin (green) immunofluorescence in the pavement cell layer, 2 µm below the surface, showing microridges of pavement cells and actin rings of apical crypts (arrowheads). (B) Same frame as A, 6 µm below the surface, showing WNK1-positive ionocytes and accessory cells. (C) An *xz*-scan showing the separation between WNK1 and actin immunofluorescence. *n*=1. Scale bar: 5 µm.

Actin is present in pavement cell microridges, and near the apical crypt of ionocytes actin forms a robust ring that likely defines the apical crypt shape (Daborn et al., 2001) and could affect other structures such as tight junctions. Actin was present in the pavement cell microridges and apical crypt rings, but did not colocalize with WNK1 in SW ionocytes (Fig. 7). Similarly, actin did not colocalize with WNK1 in ionocytes from fish acclimated to FW and 2SW (data not shown).

#### p38MAPK in SW OE

**Fig. 8.**
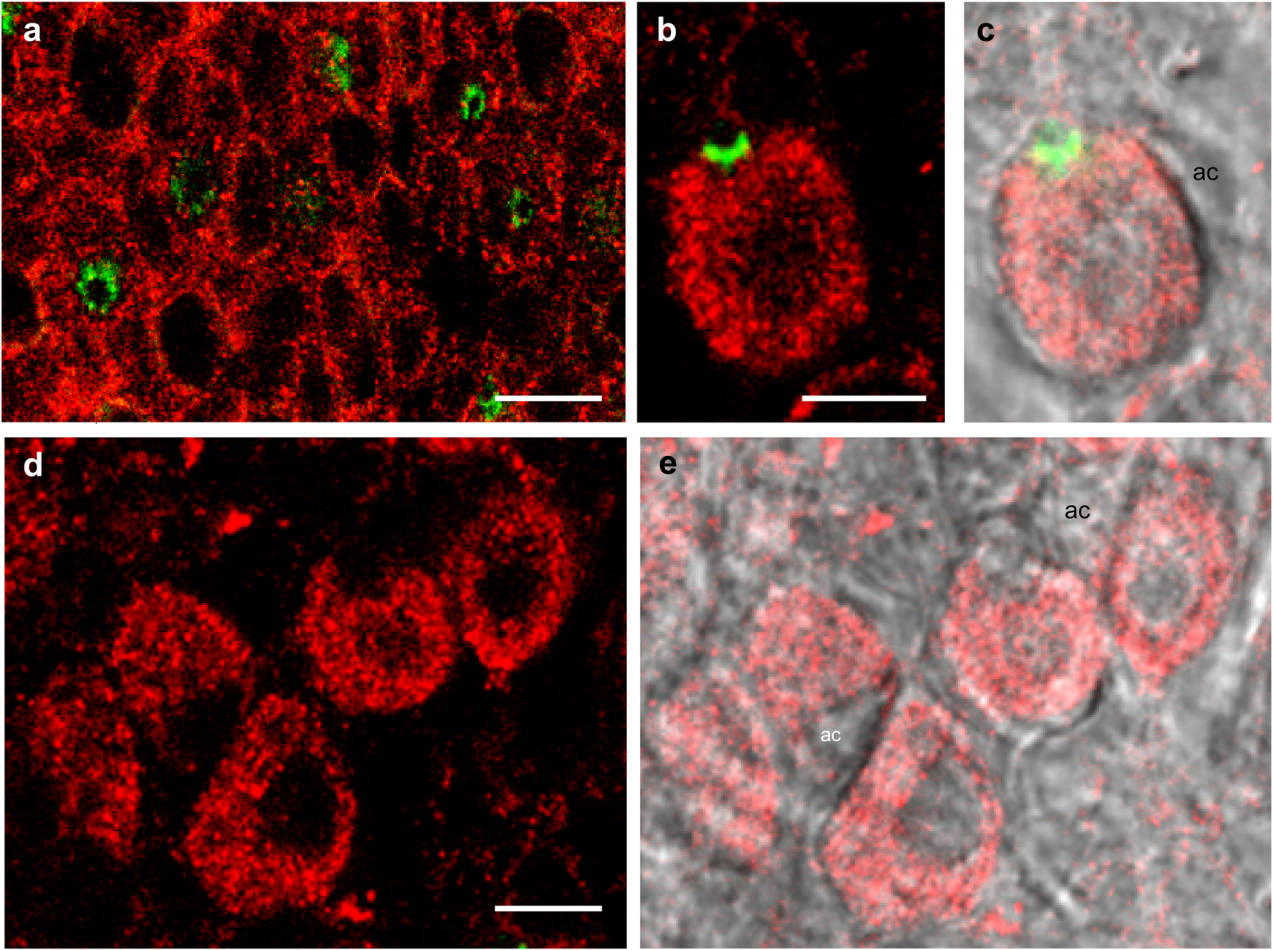
**Distribution of p38MAPK and CFTR immunofluorescence in SW OE.** (A) p38MAPK (red) was distributed in the pavement cells; CFTR (green) was restricted to the ionocyte apical crypts. (B) An *xz*-scan showing the side view of an ionocyte. (C) Same frame as B, with bright field. (D) An *xz*-scan, 12 µm below the pavement cells, shown in (E) with bright field. There was no colocalization between p38MAPK and CFTR, and p38MAPK was absent from the accessory cells. *n*=8. Scale bars: 5 µm.

**Fig. 9.**
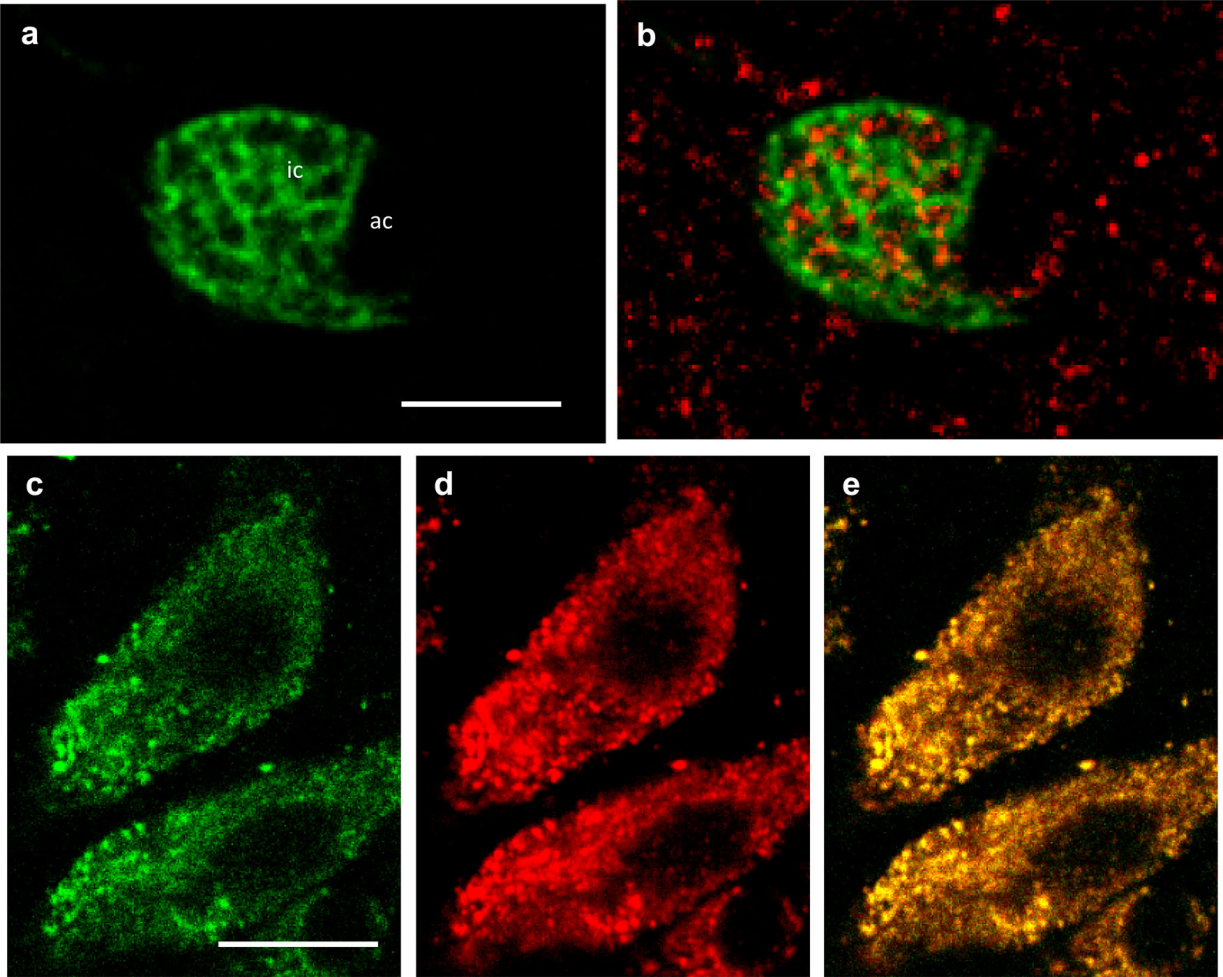
**p38MAPK and NKCC distribution in SW OE.** (A) NKCC (green) is present in the ionocytes. (B) Merged image of NKCC and p38MAPK (red) immunofluorescence showing p38MAPK distribution localized in areas where NKCC is absent. (C) Staining with MitoTracker Green (green) revealed mitochondria distribution in ionocytes. (D) p38MAPK immunofluorescence (red) in the ionocytes in C. (E) Overlay of C and D shows yellow immunofluorescence in ionocytes and colocalization of p38MAPK and Mitotracker Green. *n*=8. Scale bars: 5 µm.

The distribution of p38MAPK in SW OE included concentrated nodules throughout the pavement cells and the ionocytes (Fig. 8A-E and Fig. 9D); however, p38MAPK was absent from the accessory cells (Fig. 9C,E). Furthermore, CFTR and NKCC did not colocalize with p38MAPK (Fig. 8B and Fig. 9B, respectively) even though p38MAPK was present throughout the ionocytes (Fig. 9A). It seemed that p38MAPK was specifically localized in areas in which NKCC was absent (Fig. 9B). Merged staining of MitoTracker Green (Fig. 9C) and p38MAPK (Fig. 9D) revealed yellow immunofluorescence (i.e. colocalization), suggesting the presence of p38MAPK in the peri-mitochondrial area exclusive of the tubular system of ionocytes (Fig. 9E).

### Immunoblot

#### Protein expression of WNK1

**Fig. 10.**
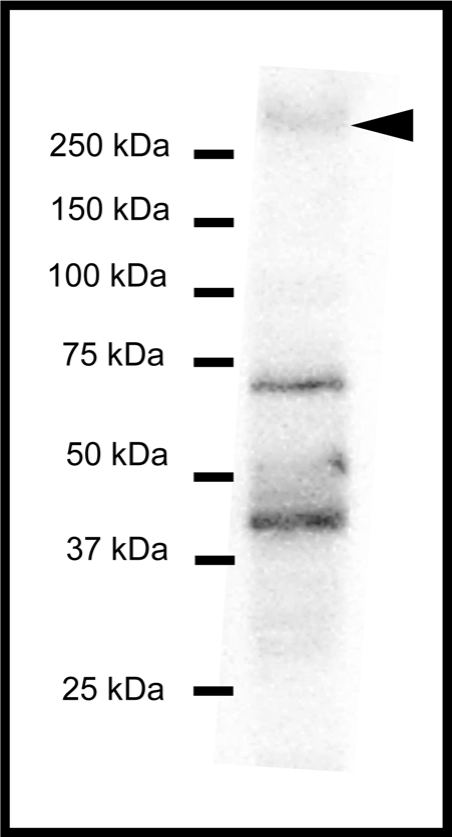
**Immunoblot showing WNK1 pT58 in SW mummichog gill tissue.** The predicted band for WNK1 (full length) was detected at ∼270 kDa (arrowhead). Non-specific bands were visible at lower molecular weights. *n*=3.

We detected WNK1 pT58 in SW mummichog gill by western blot analyses (Fig. 10, *n*=3). The antibody specific to WNK1 detected a band at ∼270 kDa and two lower molecular mass non-specific bands at ∼70 kDa and ∼40 kDa.

## DISCUSSION

The most important discovery is the strongly positive immunofluorescence of WNK1 phosphokinase in ionocytes and accessory cells of OE from fish acclimated to SW, 2SW and FW, a model tissue for the gill epithelium of teleost fish.

### WNK1 in ionocytes and accessory cells of SW and 2SW fish

WNK1 was distributed across ionocytes from fish acclimated to SW and 2SW, but did not colocalize with NKCC or CFTR. In the only previous study of WNK localization by immunocytochemistry, WNK4 has a similar distribution in mouse kidney cells; it apparently colocalizes (as viewed at low magnification) with NCC and NKCC2 but not with the junctional proteins occludin and zonulin (Ohno et al., 2011). We observed at high magnification that NKCC and WNK1 did not colocalize; rather they were within 1.0 μm of each other. In close inspection, high magnification figures indicate areas in which NCC and WNK4 are not exactly colocalized, in agreement with previously published results (Ohno et al., 2011). The background fluorescence in the OE was low, with low levels of staining in granular leucocytes (common in the epithelium) and in mucous secreting and undifferentiated cells. The strongly positive staining of accessory cells, that completely lack NKCC (or NCC), has also been observed in WNK4-positive staining of glomerulus and macula densa cells that are NKCC negative (Ohno et al., 2011). Antibody specificity is a potential issue in any immunofluorescence study, but we selected a phosphoantibody, as phosphorylation sites are more likely to be conserved across various groups. We also checked the *Fundulus heteroclitus* genome and determined that the site (-RRRHHTM-) is 100% conserved from fish to mammals and differs from the human by one residue (-RRRHTM-). Control experiments lacking the primary antibody were completely negative. There remains the remote chance that a similar phosphorylation site could exist in a completely unrelated protein, but that is true for any phosphoantibody experiment. WNK1 is known to stimulate NKCC in mammalian epithelial cells indirectly via the kinases OSR1 and SPAK (Delpire and Gagnon, 2006, 2008), so our result is consistent with this pattern, i.e. that NKCC and WNK1 are not so close as to be colocalized at the light microscope level.

Acclimation to 2SW did not apparently affect the WNK1 immunofluorescence pattern or intensity, except for a curious finding that in ionocytes from 2SW acclimated fish, there appeared a WNK-negative band immediately subjacent to the apical membrane that was significantly wider in 2SW fish than in SW fish. This area has a robust ring of actin detected by immunocytochemistry. By electron microscopy, this zone stains darker than neighboring mitochondrion-rich areas and has what appear to be protein trafficking vesicles (Cozzi et al., 2015; Hootman and Philpott, 1980; Pisam et al., 1995), hence this zone could be involved in trafficking apical membrane proteins, such as CFTR. We recently reported modification of CFTR distribution in 2SW acclimated fish and elaboration of the cation-permeable paracellular pathway (Cozzi et al., 2015). The wider gap between CFTR and WNK1 in 2SW could be to allow enhanced turnover or higher activity of CFTR.

#### Forskolin and hypertonicity

The apparent increase in WNK1-pT58 immunofluorescence in forskolin-treated tissues indicates that WNK1 may be activated in mummichog ionocytes when the cyclic AMP cascade is activated maximally. This result is consistent with WNK cascade responses, as forskolin is known to activate the WNK4-SPAK/OSR1 cascade and increase bicarbonate secretion by mouse intestine epithelium (Xiao et al., 2012) and, in HEK293 cells, forskolin increases phosphorylation at S433 and impairs degradation of WNK4 (Yoshizaki et al., 2015). Although osmotic shock can activate WNK-related cascades, we were not able to show obvious change in WNK1-pT58 signal by hypertonicity. It could be that there was a transient change that was not detected, or that hypertonic shock results in phosphorylation at a different WNK1 site. It is encouraging that a change in WNK1 phosphorylation could be demonstrated and it will be interesting to see what stressors activate this pathway in fish ionocytes.

#### WNK1 in accessory cells

The presence of WNK1 in accessory cells is intriguing, as clear functions for these cells in NaCl secretion are unknown. WNK1 was present at higher levels in accessory cells than in ionocytes, as consistently the accessory cells had two- to five-fold higher signal. The procedure could be used to specifically stain accessory cells by increasing the fluorescence threshold to eliminate the lower signal in ionocytes. This is a novel observation, as accessory cells have been known, to date, to be unstained by immunofluorescence using antibodies to several transport proteins, including CFTR and NKCC (Hiroi et al., 2005; Marshall et al., 2002b), and only weakly stained for Na^+^/K^+^-ATPase and mitochondrial stains such as MitoTracker Green (Hiroi et al., 2005; Kaneko et al., 2002; Marshall et al., 2002b; Wilson et al., 2000). The localization of the cation-selective paracellular pathway between these cells and ionocytes (Cozzi et al., 2015; Hootman and Philpott, 1980; Sardet et al., 1979) suggests that accessory cells could regulate the paracellular pathway. WNK family kinases have been associated with epithelial tight junctions. In mammalian renal epithelia, expression of WNK4 mutants produced electrophysiological changes consistent with alteration of paracellular ion (not uncharged solutes) transport, leading the authors to conclude that WNK4 is a regulator of paracellular anion permeability (Kahle et al., 2004). Thus, WNK1 presence in accessory cells could reflect WNK1 involvement in paracellular pathway permeability; in NaCl secretion, WNK1 would be involved in cation transport regulation rather than anion permeability.

### p38MAPK in ionocytes

The presence of p38MAPK in ionocytes of mummichog OE was predicted from a previous study showing activation of p38MAPK with hypertonic shock of OE, associated with regulatory volume increase and increase in transepithelial NaCl secretion (Marshall et al., 2005), using a p38MAPK antibody to the carboxy terminus of the protein, a conserved region. It is interesting that there is a complete absence of p38MAPK from accessory cells and a different distribution from WNK1 in ionocytes, still not colocalized with NKCC, but in pockets across the ionocytes and close enough to mitochondria so as to colocalize with the vital mitochondrial stain dimethylaminostyrylethylpyridinium iodide (DASPEI). Recent studies revealed that in osmotic stress responses, hyper- and hypotonic shock can increase phosphorylation of WNK at S575, and that p38MAPK can be upstream of WNK in WNK-SPAK/OSR1 cascades (Maruyama et al., 2016).

### FW ionocytes and accessory cells

Our results suggest there are several subtypes of ionocytes in FW acclimated fish. Ionocytes appear singly and often as pairs, sharing a flattened membrane surface between the two. T4 antibody immunofluorescence is present, which we assume indicates the ion uptake version of the cotransporter NCC, rather than NKCC1, although T4 antibody highlights NKCC1, NKCC2 and NCC nonspecifically (Lytle et al., 1995). To conclude unarguably that NCC is present will require specific confirmation, via qPCR or a custom antibody against mummichog NCC. WNK1 immunofluorescence varies and is absent from some single ionocytes and ionocyte pairs and, similar to the SW results, WNK1 is not colocalized with transporters (CFTR, NKCC and NCC). In FW teleost species, several types of patterns have emerged and FW teleost fish do not all share the same strategy for ion uptake. In zebrafish (*Danio rerio*, Hamilton, 1822), acid-secreting (with Na^+^ uptake and ammonia excretion), base-secreting (Cl^−^ uptake) and calcium-transporting subtypes exist and these stenohaline FW fish can live in ion poor and acidic conditions (Kumai and Perry, 2011; Kumai et al., 2011). In medaka (*Oryzias latipes*, Temminck and Schlegel, 1846), a species with more euryhaline capabilities, there are three subtypes of ionocytes in gill and OE: one that is Na^+^H^+^ exchanger-rich, a second that is NCC positive in the apical membrane with vesicle-type proton ATPase (V-ATPase) in the basolateral membrane, and a third that is positive for epithelial calcium channel (Hsu et al., 2014; Kang et al., 2015). Comparing FW teleost fish species, rainbow trout (*Oncorhynchus mykiss*, Walbaum, 1792) have acid-secreting Na^+^ uptake cells that are do not bind peanut agglutinin (PNA), designated PNA−, and α-type and base-secreting Cl^−^ uptake cells (PNA+, β-type) with epithelial calcium channel-positive apical membrane on both subtypes (Dymowska et al., 2012). The estuarine mummichog has some active Na^+^ uptake across gills and OE (Marshall et al., 1997; Wood and Marshall, 1994), but apparently no significant Cl^−^ uptake (Dymowska et al., 2012; Marshall et al., 1997; Patrick et al., 1997), instead apparently relying on dietary sources of Cl^−^ (Patrick et al., 1997). The consequence is that mummichogs have NCC-rich Na^+^ uptake cells with basolaterally located V-type H+-ATPase (Dymowska et al., 2012; Katoh et al., 2003). Because most of the FW mummichog ionocytes that are NCC positive are also WNK1 positive, it is possible that WNK1 is a regulator of NCC. Finally, the WNK1+ (NCC negative) small accessory cells in FW ionocyte pairs are a curiosity, not observed previously.

## MATERIALS AND METHODS

### Animals

Adult mummichogs (*Fundulus heteroclitus*) of both sexes (9-10 g mass) were trapped in Ogdens Pond, Antigonish County, Nova Scotia, Canada in June and transported in coolers containing estuary water to the St. Francis Xavier University Animal Care Facility. The fish were placed in full strength seawater (30.0‰ salinity) in 450 liter recirculating tanks at room temperature (20±1°C), adjusted to an ambient photoperiod under artificial lighting, and were held for several weeks prior to experimentation. Fifteen fish were acclimated for 2 weeks to hypersaline conditions (2SW=60‰ salinity), made hypersaline to seawater by adding artificial sea salt (Instant Ocean, Blacksburg, USA). Thirteen fish were transferred to dechlorinated Antigonish tap water FW and acclimated for two weeks before experimentation. Fish were fed Nutrafin flakes (R.C. Hagen, Montreal, Quebec, Canada) twice daily, so that each fish consumed 1.0 g of food per 100 g of body weight per day. Fish were also fed meal-worms (*Tenebrio molitor*) 3 days a week. They were euthanized by single pithing followed by decapitation. OE were dissected and bathed in modified Cortland's saline (composition in mmol/ l^−1^: NaCl 159.9, KCl 2.55, CaCl_2_ 1.56, MgSO_4_ 0.93, NaH_2_PO_4_ 2.97, NaHCO_3_ 17.85 and glucose 5.55, bubbled with a 99% O_2_/1.0% CO_2_ gas mixture, pH 7.7-7.8, osmolality 317 mOsm/kg). Animal care and treatment occurred under approval 16-003-N by St Francis Xavier University Animal Care Committee, following Canadian Council on Animal Care Guidelines.

### Immunocytochemistry

OE were dissected excluding the underlying dermal chromatophore layer and pinned to modeler’s sheet wax. Preparations were rinsed three times in rinsing buffer comprising 0.1% bovine serum albumin (BSA) in 0.05% Tween 20 in phosphate-buffered saline (PBS, composition in mmol l^−1^: NaCl 137, KCl 2.7, Na_2_HPO_4_ 4.3, and KH_2_PO_4_ 1.4 at pH 7.4). OE were fixed for 3 h at −20°C in 80% methanol/20% dimethyl sulfoxide (DMSO), then rinsed and immersed in a blocking solution with 5% normal goat serum, 0.1% BSA, 0.2% NaN_3_ in TPBS, pH 7.4 for 30 min at room temperature in the dark. For mitochondria detection, OE were incubated in 200 nmol l^−1^ MitoTracker Green FM (Thermo Fisher Scientific) in PBS for 2 h at room temperature in the dark, prior to fixation (Marshall et al., 2002a). The OE were then incubated in the primary antibodies (10 µg/ml in blocking solution) at 4°C overnight. Following three (5 min) rinses in PBS, the OE were exposed to the secondary antibodies (8 µg/ml in blocking solution) for 4 h at room temperature in the dark. After three final rinses in PBS, the OE were mounted in a mounting medium (Fluoroshield™, Sigma-Aldrich) and slides were viewed in a single blind fashion. Images were collected with a laser scanning confocal microscope (FV300, Olympus, Markham, Canada). In each OE, fields were randomly selected and *z*-stack series were collected using a 60× water objective (N.A. 1.20W), zoom of 3.0 and optical sections of 0.50 µm. An average of 30 sections was collected for each image. Perspective 3D surface plots were obtained using ImageJ 1.48v image processing program (NIH).

### CFTR and WNK1 gap

Analysis of WNK1 proximity to the apical crypt in SW and 2SW OE (measurements were taken from *z*-stack images 2 µm below the surface; *n*=4 epithelia, eight *z*-stack images and 90 cells in total). CFTR and WNK1 distribution measurements were calculated in a single blind fashion; areas were selected at random. At 2 µm below the surface of the epithelium, apical crypts were identified by CFTR immunofluorescence, and WNK1 exclusion zone distances were measured for all ionocytes within the chosen area.

### Protein extraction

Gill filaments from four SW mummichogs were dissected and pooled. Gill tissue was used because this is the main (and larger) osmoregulatory organ rather than the accessory OE. The minced tissue was transferred to lysis buffer [50 mM Tris-HCl, pH 7.5, 150 mM NaCl, 1% sodium deoxycholate, 1% Triton X-100, 0.1% sodium dodecyl sulfate (SDS), 1 mM phenylmethylsulfonylfluoride, protease inhibitor cocktail (Sigma-Aldrich)] and homogenized with a Polytron homogenizer (Brinkmann Polytron PT-3000, Brinkmann Instruments Mississauga, Canada). The lysate was centrifuged at 20,000 ***g*** for 20 min at 4°C). Supernatant was collected and protein concentration was determined by the Bradford method. Protein extracted (30 µg) was then mixed with 5× SDS-sample buffer (100 mM Tris/HCl pH 8.0, 4.8% SDS, 16% glycerol, 0.1% Bromophenol Blue) supplemented with 2% β-mercaptoethanol), and boiled for 5 min.

### Western blots

Proteins were separated using SDS-PAGE (precast Mini-PROTEAN^®^ TGX™ precast gels 10%, Bio-Rad) and electro-transferred to PVDF membranes (Trans-Blot^®^ Turbo™ Transfer System, Bio-Rad). Following transfer, the membranes were incubated in blocking buffer [Tris-buffered saline plus 0.05% Tween 20 (TTBS), supplemented with 1% BSA] for 1 h at room temperature. Primary antibodies were diluted to a concentration of 1 µg/ml in blocking buffer and applied overnight at 4°C. The blots were washed three times (10 min) in TTBS. The horseradish peroxidase-conjugated secondary antibody was diluted 1:100,000 to a concentration of 12.5 ng/ml in blocking buffer (TTBS supplemented with 2% BSA), and applied for 1 h at room temperature. The blots were then washed three times (15 min) in TTBS. Immunoreactive bands were detected and visualized by chemiluminescence using Clarity™ Western ECL substrate (Bio-Rad) and ChemDoc™ MP Image System (Bio-Rad).

### Antibodies

The primary antibody used for detection of mummichog CFTR was mouse monoclonal anti-human CFTR (MAB25031, clone 24-1, R&D Systems, Minneapolis, USA) with the epitope at the carboxy terminus, a zone that is conserved in mummichog to human (Singer et al., 1998, 2008) and therefore is selective for this protein (Marshall et al., 2002b; Robertson and Hazel, 1999). The primary antibody used for detection of NKCC was T4, an antibody to the carboxy region of NKCC that has been shown to bind to several isoforms of NKCC across several species (Lytle et al., 1992; Wilson et al., 2000). T4 (DSHB Hybridoma Product T4) was deposited to the Developmental Studies Hybridoma Bank (University of Iowa, Iowa City, USA). The primary antibody used to detect WNK1 was a phospho T58 affinity purified rabbit polyclonal antibody (phosphopeptide derived from human WNK1 around the phosphorylation site of threonine 58 (ab53137, Abcam). MAPK p38 was detected by affinity purified rabbit polyclonal anti-P38 (C20) primary antibody (CLDB022, Cedarlane, Burlington, Canada). The secondary antibodies used for immunofluorescence microscopy were goat anti-rabbit immunoglobulin and goat anti-mouse immunoglobulin, conjugated with Alexa Fluor 488 (A11001) and Alexa Fluor 546 (A-11003), respectively (Life Technologies). Negative controls were performed by omitting the primary antibody. Actin immunofluorescence was detected using JLA20 mouse monoclonal antibody. JLA20 (DSHB Hybridoma Product JLA20) was deposited to the Developmental Studies Hybridoma Bank.

### Statistical analyses

Data are expressed as mean±s.e.m. The significance of differences in WNK1 distance from the apical crypt was determined by unpaired two-tailed *t*-test. *P*<0.05 was considered statistically significant.
